# RASSF2 Under the Computational Microscope: Prediction of Destabilizing Missense Variants

**DOI:** 10.3390/biology15201818

**Published:** 2026-10-12

**Authors:** Laith Naser AL-Eitan, Mohammad Tareq Abusalem, Mansour Abdullah Alghamdi

**Affiliations:** 1 Department of Biotechnology and Genetic Engineering, Jordan University of Science and Technology, Irbid 22110, Jordan; mtabusalem22@sci.just.edu.jo; 2 Department of Anatomy, College of Medicine, King Khalid University, Abha 61421, Saudi Arabia; m.alghamdi@kku.edu.sa; 3 Genomics and Personalized Medicine Unit, The Center for Medical and Health Research, King Khalid University, Abha 62529, Saudi Arabia

**Keywords:** RASSF2, tumor suppressor gene, variants, cancer, MutPred-PPI, AlphaFold3

## Abstract

Background: RASSF2 is a tumor suppressor gene involved in multiple cancers such as colorectal and lung cancer. Different studies have focused on promoter hypermethylation as the main inactivation pathway of this gene, which leads to cancer proliferation and metastasis. Here, using a comprehensive in silico pipeline, we investigate, for the first time, the impact of missense variants on protein structure, stability, and interactions. Methods: Multitiered computational tools were used to predict the most likely pathogenic and destabilizing nsSNPs; the impact of coding mutations on different gene expression was analyzed, and the impact of nsSNPs on PPIs using mCSM-PPI2 and MutPred-PPI was analyzed using predicted AlphaFold protein complexes. Results: Of 454 variants, 19 variants were consistently predicted to be likely pathogenic and destabilizing, 14 variants were in the RA domain, and two variants were in the SARAH domain. V193D, L235P, L247P, and Y275C were conserved and exhibited severe structural and molecular alterations. Also, Y275C was at the interface, and it was predicted to disrupt the RASSF2-MST2 complex. KRT23 and BCL2L14 were significantly downregulated in samples containing mutated RASSF2 in COAD and LUAD, respectively. STRING illustrates the Hippo signaling pathway as the main pathway involved in the network. Conclusions: These findings provide promising candidates that can be important for future functional investigations. However, in vivo and in vitro experiments to validate our findings are necessary.

## Simple Summary

The inactivation of the RASSF2 gene has been implicated in multiple types of cancer, and predicting variants that could potentially lead to its destabilization is important. Here, we used, for the first time, comprehensive machine-learning methods to prioritize the most destabilizing nsSNPs on the protein structure and function. Our study reports missense variants that can potentially alter the structure, function, and interactions of RASSF2. These variants may serve as candidates for future functional investigations.

## 1. Introduction

Ras association (RalGDS/AF-6) domain family member 2 (RASSF2) is a tumor suppressor gene located at 20p13. It is important in cell cycle control and microtubule stabilization [1]. RASSF2 interacts with the KRAS protein via its effector domain, thus acting as a negative effector [2], and it travels to the nucleus, where it performs its apoptotic function [3]. RASSF2 is mainly inactivated through hypermethylation of the promoter in colorectal, lung, gastric, breast, and thyroid cancers [3,4,5,6].

RASSF2 is similar in structure to RASSF1A, and it acts as a KRAS effector protein that is found in complex with KRAS [7]. In fact, evidence has emerged that points to RASSF2 being the strongest RAS death effector, since there is a positive correlation between inactive RASSF2 and mutations in KRAS/BRAF in tumors [8,9]. RASSF2 acts as a tumor suppressor gene, in which its overexpression promotes apoptosis and cell cycle arrest in vitro and inhibits tumor growth in vivo [2,6], and loss of its expression promotes invasion, growth, and transformation [7,9].

In addition to its binding with KRAS, RASSF2, through its Sav/Rassf/Hpo (SARAH) domain, binds two kinases, mammalian sterile STE20-like kinase 1 or serine/threonine kinase 4 (MST1, STK4) and mammalian sterile STE20-like kinase 2 or serine/threonine kinase 3 (MST2, STK3), forming an active complex in vivo. RASSF2 is kept in its phosphorylated state to prevent degradation of MST2 [10]. Thus, loss of RASSF2 in tumors severely weakens the apoptotic activity. Non-synonymous single nucleotide polymorphisms (nsSNPs) are the main contributors to around 50% of mutations related to various genetic diseases [11,12]. Damaging impact on the protein structure and function can result from nsSNPs, as they can cause alterations in protein charge, geometry, and hydrophobicity [13]. Moreover, they can weaken protein–protein interactions (PPIs) [14]. Thus, it is important to investigate missense variants that might impact this interaction.

Investigating the structural and molecular effects of nsSNPs using experimental methods is costly and time-consuming. Recently, computational prediction of damaging nsSNPs has emerged as a useful tool to discriminate between damaging nsSNPs and neutral ones by prioritizing the most likely damaging nsSNPs for clinical studies. Using consistency across tools and a combination of these different algorithms increases the accuracy, precision, and performance of such tools [15]. In addition, structural prediction tools have been increasingly used for cancer variant prioritization, due to the implementation of new state-of-the-art tools such as AlphaFold 3 and AlphaMissense [16,17].

Therefore, this study aims to predict and characterize the most likely pathogenic, destabilizing, and oncogenic nsSNPs on the structure, function, and interaction of the RASSF2 gene using integrative in silico methods. Predicting damaging nsSNPs in this gene will pave the way for a better understanding of the impact of these genetic variants on the protein structure and function, and will provide a hypothesis that can be useful in the field of pharmacogenomics. To our knowledge, this is the first study that addresses nsSNPs in the RASSF2 gene.

## 2. Materials and Methods

### 2.1. SNP Data and Protein Sequence Retrieval

We utilized the National Center for Biotechnology Information (NCBI) dbSNP (https://www.ncbi.nlm.nih.gov/snp/) (accessed on 6 August 2026) to retrieve all the nsSNPs of the RASSF2 gene (Gene ID: 9770) relative to the MANE transcript (NM_014737.3) [18]. Multiallelic rsIDs were treated as separate missense variants when they have different amino acid changes. The protein sequence of RASSF2 in FASTA format was retrieved from UniProt, prioritizing the reference sequence: P50749 [19]. Only the missense or non-synonymous SNPs (nsSNPs) were utilized for our in silico study.

### 2.2. Predicting the Most Deleterious nsSNPs

The Sorting Intolerant from Tolerant (SIFT) (https://siftdna.org/) (accessed on 7 August 2026) is a web server that predicts the functional effects of amino acid substitutions by utilizing sequence homology and physicochemical properties of amino acids [20]. Default parameters and missense variants in the required format were submitted to the server. A Tolerance Index (TI) value < 0.05 indicates a deleterious mutation, whilst a value ≥ 0.05 indicates a tolerated mutation.

The Polymorphism Phenotyping v2 (PolyPhen2) (https://genetics.bwh.harvard.edu/pph2/index.shtml) (accessed on 7 August 2026) is a computational tool that predicts the potential impact of a missense mutation on the structure and function of a human protein, based on simple physical and comparative considerations [21]. PolyPhen2 calculates sensitivity, specificity, and the PSIC (position-specific independent count) score. Default parameters and missense variants in the required format were submitted to the server. The numerical score ranges from 0.0 (benign) to 1.0 (probably damaging). The tool categorizes SNPs as probably damaging, possibly damaging, or benign.

PhD-SNP^g^ (https://snps.biofold.org/phd-snpg/) (accessed on 8 August 2026) is a binary classifier for coding and non-coding variants for predicting pathogenic human SNPs [22]. This tool was trained using ~104,000 deleterious and neutral SNPs from the ClinVar database. Missense variants in the required format using default parameters were submitted. A score > 0.5 indicates a predicted pathogenic SNP.

E-SNPs&GO (https://esnpsandgo.biocomp.unibo.it/) (accessed on 8 August 2026) is a machine-learning method for predicting the pathogenicity of human genetic variations. It classifies input variants as either pathogenic or benign by applying a support vector machine (SVM) classifier [23]. E-SNPs&GO uses two different protein embedding schemes: ESM-1v (an encoder-only model), ProtT5 (a sequence-to-sequence model), and Gene Ontology (GO) functional annotations. A calibrated probability ≥ 0.5 indicates a predicted pathogenic variation. E-SNPs&GO was used under default parameters.

AlphaMissense, a state-of-the-art tool, is an adaptation of AlphaFold fine-tuned on human and primate variant population frequency databases to predict the pathogenicity of missense variants [24]. A score > 0.5 indicates a likely pathogenic variant.

Variants predicted to be pathogenic by all five tools were prioritized for downstream structural stability analysis.

### 2.3. Protein Three-Dimensional Structure Prediction and Assessment

The AlphaFold Protein Structure Database (https://alphafold.ebi.ac.uk/) (accessed on 10 August 2026) tool was used to retrieve the three-dimensional (3D) structure of the RASSF2 protein (structure ID: AF-P50749-F1; version 6). This tool contains millions of highly accurate protein structure predictions [25]. The tool provides a confidence measure (pLDDT, or predicted Local Distance Difference Test) on a scale of 0 to 100, with higher values indicating greater reliability. It also generates a pTM value (predicted template modelling), which reflects the overall accuracy of the predicted structure. Regions with a pLDDT value above 90 are considered very reliable; values between 70 and 90 indicate moderate reliability, values between 50 and 70 indicate low reliability, and values below 50 indicate very low reliability. Furthermore, a pTM value above 0.5 indicates that the predicted overall conformation (folding) of the complex corresponds to the actual structure. The UniProt ID (P50749) was used as an input to retrieve the 3D structure corresponding to the canonical UniProt ID (P50749) sequence. To assess the overall structural quality of the AlphaFold prediction, the built model was assessed using the ERRAT and PROCHECK Ramachandran plot analysis tools accessed via the SAVES v6.1 web server (https://saves.mbi.ucla.edu/) (accessed on 10 August 2026). ERRAT assesses the overall quality of the model based on non-covalent atomic interactions [26]. PROCHECK assesses the stereochemical quality of the protein structure; it shows the distribution of residues in favored, allowed, and disallowed regions [27]. Additionally, we used AlphaFold 3 to prepare two complexes for the RASSF2 protein: RASSF2—GTP-bound KRAS and RASSF2-MST1, for downstream variants’ impact on PPIs. For RASSF2—GTP-bound KRAS, the RA domain of the RASSF2 sequence was used as an input along with the active KRAS sequence that includes guanosine triphosphate (GTP) and magnesium ions (Mg^2+^), since this is the active state that binds effectors. For RASSF2-MST1, the SARAH domain sequence of RASSF2 and the SARAH domain sequence of MST1 were used as inputs in the AlphaFold 3 server. Seeds were set to auto, resulting in five generated models per run, the model with the highest metrics (pTM and ipTM) was selected. In addition, multiple independent runs were conducted with the same parameters, which revealed that all predicted complexes had consistent interfaces.

As for RASSF2-MST2, we retrieved the deposited complex in the AlphaFold database covering the full length of RASSF2 and MST2 (AF-0000000210953970, version v1, NVIDIA dataset). For RASSF2-KRAS and RASSF2-MST1 complexes, plDDT, pTM, interface predicted template modelling (ipTM), and predicted alignment error (PAE) plot were assessed to understand the model confidence. PAE reflects the predicted error between relative positions of residue pairs. ipTM measures the accuracy of the predicted relative positions of the subunits within the complex. Values > 0.8 reflect confident predictions, while values below 0.6 suggest a likely failed or low-confidence prediction, and ipTM values between 0.6 and 0.8 are a gray zone where predictions could be correct or incorrect or of medium confidence. As for the RASSF2-MST2 complex, plDDT, ipTM, interaction prediction score from aligned errors (ipSAE), and pDockQ2 were assessed to understand model confidence. ipSAE focuses on inter-chain residue pairs with more reliable, predicted positioning, and pDockQ2 assesses the expected quality of an interface by combining local residue confidence with confidence in the predicted relative positioning across the interface. A very confident score (ipSAE ≥ 0.8) indicates that the interface is modelled with high accuracy. A confident score (0.8 > ipSAE ≥ 0.7) indicates the predicted interface is likely correct with a well-arranged interaction. A low confidence score (0.7 > ipSAE ≥ 0.6) indicates the interaction may be correct but should be interpreted cautiously. A pDockQ2 value above ~0.23 indicates a likely acceptable interface.

### 2.4. Predicting the Effect of Variants on Protein Stability

To predict the impact of nsSNPs on protein stability, three computational methods were utilized:

DDMut (https://biosig.lab.uq.edu.au/ddmut/) (accessed on 10 August 2026) is a deep learning method that can predict changes in Gibbs Free Energy (ΔΔG) upon single or multiple point mutations [28]. DDMut uses optimized graph-based signatures with other physicochemical properties to generate its output. A score < 0 indicates a predicted destabilizing variant, while a score > 0 indicates a stabilizing variant. The protein structure in the Protein Data Bank (PDB) format and the list of variants were used as input.

DynaMut2 (https://biosig.lab.uq.edu.au/dynamut2/) (accessed on 10 August 2026) was used to assess changes in protein stability and flexibility upon missense mutations. This tool predicts the changes in energy and vibrational entropy between the mutant and wild-type proteins [29]. Like DDMut, a negative score indicates a predicted destabilizing variant. The protein structure in PDB format and the list of variants were used as input.

ThermoMPNN (https://app.tamarind.bio/tools/thermompnn) (accessed on 10 August 2026) is a deep learning method that can predict the impact of amino acid variations on protein stability efficiently and rapidly [30]. This tool achieves state-of-the-art prediction on different benchmark datasets. The protein 3D structure was used as an input, and the result shows changes in Gibbs free energy (ΔΔG) for each variant; ΔΔG > 0 indicates a predicted destabilizing variant, while ΔΔG < 0 indicates a predicted stabilizing variant.

Only variants that have ΔΔG < −0.5 in DDMut and DynaMut2, and ΔΔG > 0.5 in ThermoMPNN, across the three tools were prioritized for downstream analysis. We justify this by recent evidence that protein stability predictor algorithms have unreliable results when the mutation is predicted to cause a ΔΔG within the interval ± 0.5 kcal/mol [31].

### 2.5. Population Allele Frequency Retrieval

Genome Aggregation Database (gnomAD v4.1.1) (https://gnomad.broadinstitute.org/) (accessed on 11 August 2026) was used to retrieve the population allele frequency of our most destabilized variants [32]. This is a resource for aggregating sequencing data from the exome and genome. We submitted the gene name, and we manually pinpointed the minor allele frequencies (MAF) for each SNP in different ethnic groups. In addition, clinical annotations for RASSF2 variants were investigated using the ClinVar database (https://www.ncbi.nlm.nih.gov/clinvar/) (accessed on 11 August 2026), a publicly accessible database, updated weekly, which contains interpretations of the clinical significance of human gene variants, compiled on the basis of analyses carried out by various clinical and research institutions [33].

### 2.6. Conservation Profile Using ConSurf Tool

The ConSurf web server (https://consurf.tau.ac.il/) (accessed on 11 August 2026) was used to determine the evolutionary conservation of variant positions in the RASSF2 protein sequence [34]. This tool provides a conservation score ranging from 1 to 9, where a score of 9 indicates a highly conserved position and a score of 1 indicates a highly variable position. The PDB format of RASSF2 was submitted to the server using default parameters, and we screened for the conserved residues.

### 2.7. Identification of RASSF2 Protein Domains

The RASSF2 protein sequence was submitted to the InterPro server (https://www.ebi.ac.uk/interpro/) (accessed on 11 August 2026). This tool predicts the protein family and conserved domains. We manually located the nsSNPs within these domains [35].

### 2.8. Prediction of Surface Accessibility and Secondary Structure

NetSurfP-3.0 (https://services.healthtech.dtu.dk/services/NetSurfP-3.0/) (accessed on 13 August 2026), a state-of-the-art tool, was used to explore the surface accessibility (buried or exposed), secondary structures (SS), and probability of disorder (Pdisorder) for the wild-type RASSF2 protein and its mutant variants [36]. The wild-type protein sequence and mutant protein sequences were submitted individually to the server with default parameters. The tool generates relative surface accessibility (RSA), absolute surface accessibility (ASA), Pdisorder for each amino acid, and phi/psi dihedral angles for each amino acid. All the prioritized variants were analyzed individually to gain insight into the differences between the wild-type and mutant protein in terms of SS, surface accessibility, and changes in disorder.

### 2.9. Prediction of Variants Association with Cancer

Somatic variant oncogenicity classification relies on consensus frameworks such as the Clinical Genome Resource (ClinGen), Cancer Genomics Consortium (CGC), and Variant Interpretation for Cancer Consortium (VICC) guidelines [37]. We assessed potential oncogenic variants from the identified germline missense variants using CScape (https://cscape.biocompute.org.uk/) (accessed on 13 August 2026). CScape is an integrative classifier that relies on statistical approaches to predict cancer driver mutations. A mutation list in the required input was used for all the prioritized variants [38]. The analysis utilized GRCh38/hg38 as the reference genome assembly; we entered the variants in the specified format: chromosome, position, reference base, mutant base. A score > 0.5 indicates a predicted oncogenic variant, while a score higher than 0.9 indicates a high confidence level in predicting potential oncogenic variants. CScape metrics served only as a risk scoring tool rather than definitive proof of somatic transformation. Furthermore, we used CBioportal and AACR Project GENIE to investigate whether the specific prioritized variants or their specific codon positions harbor somatic alterations in clinical cohorts. We used the RASSF2 gene as a query in both databases.

CBioportal (https://www.cbioportal.org/) (accessed on 13 August 2026), which is a web server that contains various data on the molecular landscape of multiple types of cancer [39]; AACR Project GENIE is an international data-sharing consortium whose aim is to establish an evidence base for precision oncology. This is achieved by combining clinical-grade genomic data on cancer with data on clinical treatment outcomes from tens of thousands of cancer patients treated at numerous centers around the world [40].

### 2.10. Prediction of Phosphorylation Sites

To predict phosphorylation sites in the RASSF2 protein, NetPhos3.1 (https://services.healthtech.dtu.dk/services/NetPhos-3.1/) (accessed on 13 August 2026) and GPS 6.0 (https://gps.biocuckoo.cn/online.php) (accessed on 13 August 2026) were used. NetPhos3.1 can predict serine, threonine, and tyrosine phosphorylation sites in human proteins using ensemble neural networks [41]. The RASSF2 amino acid sequence was used as an input, and the results show a score from 0–1; a score > 0.5 indicates a positive prediction. GPS 6.0 predicts protein kinase (PK)-specific phosphorylation sites utilizing logistic regression, deep neural network, and light gradient boosting machine [42]. The RASSF2 amino acid sequence was used as an input; a score > 0.76 indicates a predicted phosphorylated site in GPS 3.0.

### 2.11. Predicting Functional and Molecular Effects of the nsSNPs

We utilized the MutPred2 tool (https://mutpred.mutdb.org/index.html) (accessed on 14 August 2026) to predict the pathogenicity of the variants and their molecular mechanisms. MutPred 2 is an effective machine learning-based web server that predicts the pathogenicity of missense variants and, at the same time, their impact on more than 50 different protein properties [43]. This tool assigns variants a value (g-score) on a scale from 0 to 1 based on the probability that a specific amino acid variant causes disease. The g-score corresponds to the average of all sub-scores determined by the neural networks of the MutPred2 tool. The RASSF2 amino acid sequence was used as an input using default parameters. A probability value > 0.5 indicates that the missense variant is a predicted pathogenic variant, whilst a value < 0.5 indicates a non-pathogenic variant. A value of 0.68 corresponds to a 10% false-positive rate, whilst a value of 0.80 represents a 5% false-positive rate; thus, the higher the g-score, the greater the likelihood of pathogenicity. The RASSF2 protein sequence and the prioritized variants were submitted to this server.

### 2.12. Prediction of the Structural Effect Using Project HOPE

The impact of missense variants on the structure and function of the RASSF2 protein was predicted using the Have (y) Our Protein Explained (Project HOPE) server (https://www3.cmbi.umcn.nl/hope/) (accessed on 14 August 2026) [44]. This tool was developed to characterize the effects of variants in human proteins on the molecular basis of disease-associated phenotypes. The sequence of the RASSF2 protein was entered into the system, together with information on the wild-type amino acids and the mutated variants. The results provided a prediction of the effect of the specific amino acid variant on the protein structure, as well as on the physicochemical properties resulting from the amino acid substitution.

### 2.13. Prediction of the Impact of Variants on PPIs

To predict the impact of variants on PPIs, mCSM-PPI2 and MutPred-PPI were used. mCSM-PPI2 (https://biosig.lab.uq.edu.au/mcsm_ppi2/) (accessed on 14 August 2026) is a novel machine learning tool that can accurately predict the impact of mutations on PPI binding affinity by using graph-based structural signatures [45]. Each predicted complex was submitted along with variants related to its position. The tool provides a predicted affinity change, where values < 0 indicate a decrease in binding affinity, while values > 0 indicate an increase in binding affinity. Also, it provides a value for distance from the closest partner, where a residue is at the interaction interface if it is located at most 5 angstroms (Å) away from the interacting partner. MutPred-PPI v1.0 (https://mutpred.mutdb.org/mutpredppi/) (accessed on 14 August 2026) is a graph attention network that can effectively predict interaction-specific (edgetic) effects of missense variants by utilizing AlphaFold 3-based protein complex contact graphs with protein language models (pLMs) embeddings imposed upon nodes [46]. MutPred-PPI achieves an area under the curve (AUC) of 0.85 on seen proteins and an AUC of 0.72 on unseen proteins, achieving strong generalizability despite the limited training data. The three predicted complexes were submitted independently, along with variants related to the specific domain. The tool generated a score from 0–1, where a score > 0.5 indicates potentially disruptive interactions due to missense mutation, while a score < 0.5 indicates preserved interactions. ChimeraX v1.10, a next-generation molecular visualization tool, was used to visualize the closest variants to the interaction interfaces across the three protein complexes [47].

### 2.14. Analysis of the Correlation Between Gene Expression Level and Mutation in RASSF2

Using the muTarget tool (https://mutarget.com/) (accessed on 15 August 2026), we investigated the correlation between mutations and gene expression using the “genotype” run [48]. muTarget accuracy comes from statistical methods like log-rank and Fisher’s exact tests. It has high sensitivity for detecting driver variants, although false positives may occur due to data noise in high-throughput sequencing. The gene name was used as an input, and mutation type was selected for coding mutations only, and the analysis was conducted for colon adenocarcinoma (COAD) and lung adenocarcinoma (LUAD) cancer types. The muTarget database contains 24 solid tumor subtypes and over 9300 patient samples, incorporating somatic mutations and RNA-seq gene expression data. We used muTarget with default parameters such as *p*-value cutoff ≤ 0.01, fold change cutoff = 1.44, and FDR ≤ 5%.

### 2.15. Prediction of PPIs Network

The Search Tool for the Retrieval of Interacting Genes/Proteins (STRING) version 12.0 (https://string-db.org/) (accessed on 16 August 2026) was used to predict interactions between the RASSF2 protein and other proteins. STRING is an online database that compiles both confirmed and predicted protein–protein interactions. This tool integrates data from various sources indicating such interactions, including the results of experimental studies, computational methods, and text analysis from open-access sources [49]. To identify interactions involving the RASSF2 protein, the search function by protein name in the STRING database was used; entering the term ‘RASSF2’ produced a graphical representation of this protein’s interactions. A confidence score threshold of over 0.7 was set, and the maximum number of partner proteins displayed was limited to 10. The full STRING network option was chosen, in which the edges indicate both functional and physical protein associations; also, the default evidence channels were used, which include text mining, experiments, databases, co-expression, neighborhood, gene fusion, and co-occurrence. In addition, STRING was used to identify the biological processes and Kyoto Encyclopedia of Genes and Genomes (KEGG) pathways (according to the GO classification) associated with proteins that interact with RASSF2. Enrichment analysis was performed against the whole human proteome as a statistical background.

## 3. Results

### 3.1. Retrieving the nsSNPs from the NCBI Database

The NCBI dbSNP was used to retrieve the data used in this study. 19,303 SNPs were found; of them, 454 were nsSNPs (Missense SNPs), and they were retrieved for this study (Table S1). All 454 missense variants correspond to unique amino acid substitutions.

### 3.2. Prediction of Pathogenicity Using Five Tools

**Figure 1:**
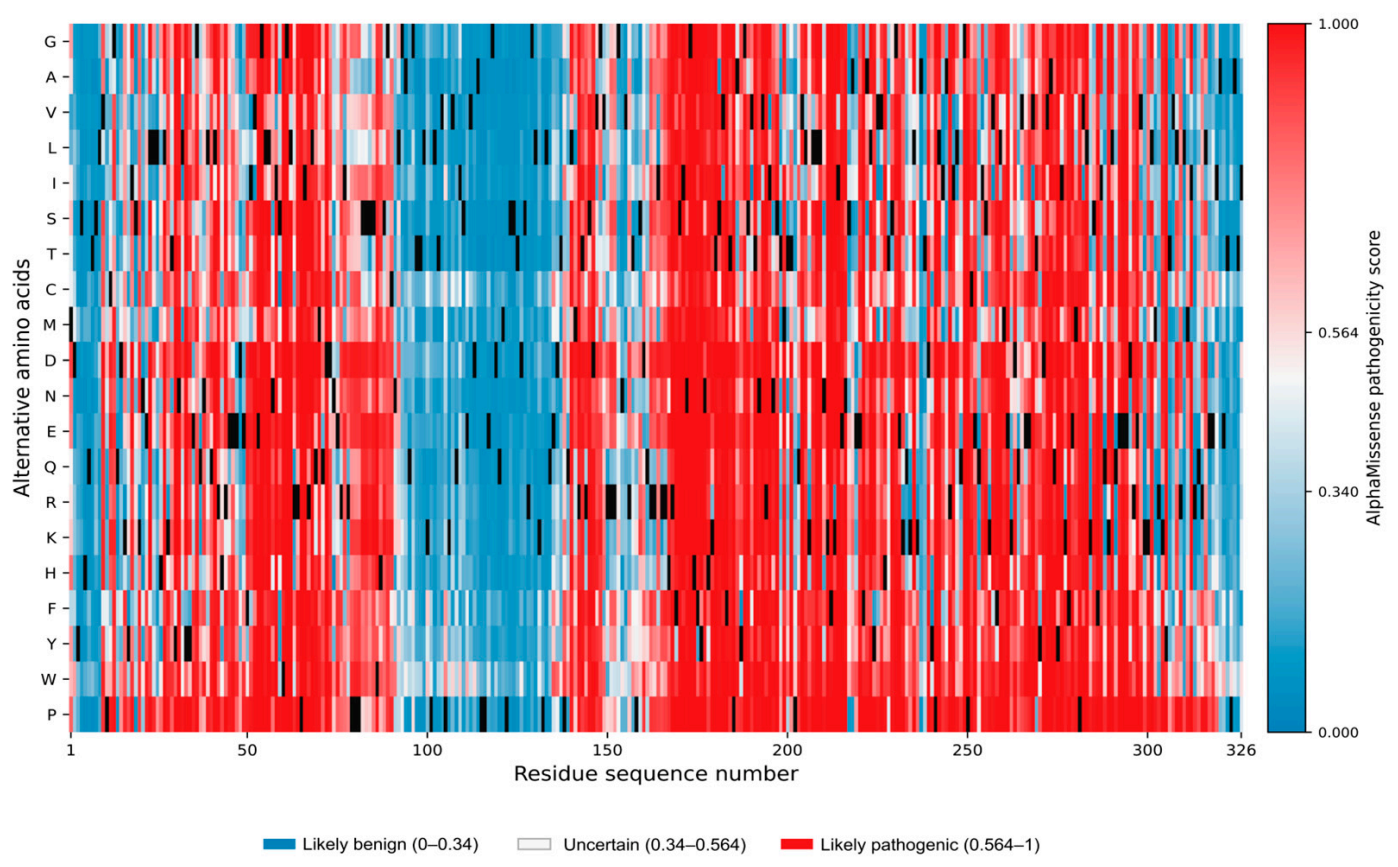
Heatmap generated by AlphaMissense indicating potential pathogenicity prediction for RASSF2 variants.

All 454 missense variants were submitted to five pathogenicity tools (SIFT, PolyPhen 2, PhD-SNP, E-SNPs&GO, and AlphaMissense). SIFT predicted 196 variants to be potentially deleterious (Table S2), PolyPhen2 predicted 297 variants to be likely damaging (Table S3), PhD-SNP predicted 301 variants to be potentially pathogenic (Table S4), and E-SNPs&GO predicted 134 variants to be probably pathogenic (Table S5), and AlphaMissense predicted 210 variants to be likely pathogenic (Figure 1; Table S6). As a result, 75 variants were predicted to be likely deleterious, damaging, pathogenic, and disease-causing by all five tools (Table S7; Table 1), and they were prioritized for structural stability analysis.

### 3.3. Prediction of the Protein 3D Structure

**Figure 2:**
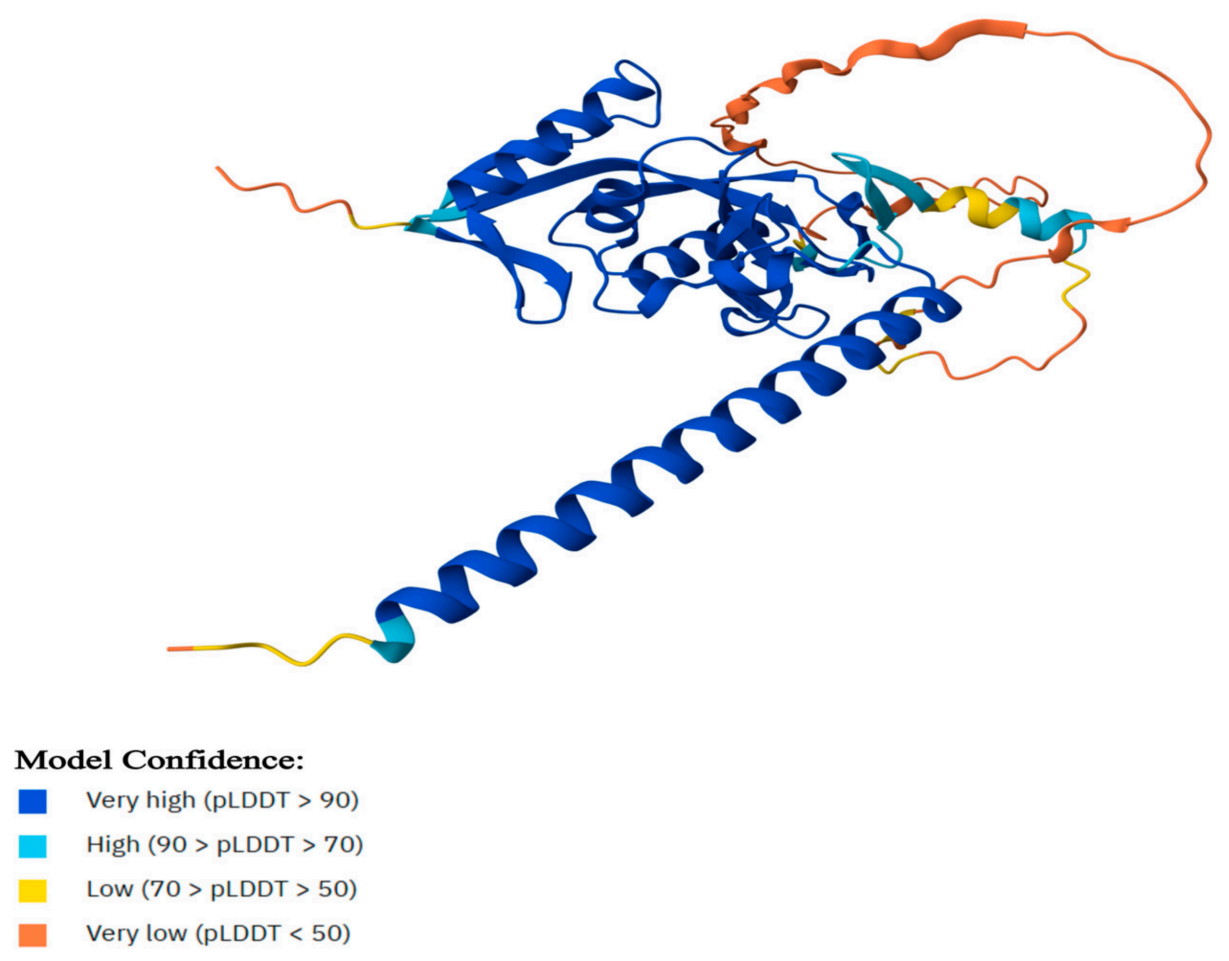
AlphaFold prediction for the RASSF2 protein 3D structure.

AlphaFold database was used to retrieve the 3D structure of the RASSF2 protein using the UniProt accession: P50749. The average pLDDT is 77.88, reflecting confident prediction (Figure 2). In addition, the PAE plot showed the RA and SARAH domains with low PAE, indicating a good relative rearrangement (Figure S1A). Most of the 75 prioritized variants were in high- and very high-confidence regions. The 3D structure was further assessed using ERRAT and PROCHECK. ERRAT illustrated an overall quality factor of 92.4%. PROCHECK showed that 83.8% of residues were placed in the Ramachandran plot’s most favored regions.

### 3.4. Predicting the Effect of Variants on Protein Stability

All 75 variants predicted to be likely pathogenic were submitted to DDMut, DynaMut2, and ThermoMPNN. DDMut predicted 51 variants to decrease stability (∆∆G < 0) (Table S8); DynaMut2 predicted 54 variants to decrease stability (∆∆G < 0) (Table S9); ThermoMPNN, on the other hand, predicted 64 variants to decrease stability (∆∆G > 0) (Table S10). As a result, 19 variants were consistently predicted to decrease the stability of the RASSF2 protein across all three tools (∆∆G < −0.5 in DDMut and DynaMut2; ∆∆G > 0.5 in ThermoMPNN) (Table 2 and Table S11). These 19 variants were prioritized for in-depth analysis.

### 3.5. Population MAF Associated with the Prioritized Variants

gnomAD v4.1.1 database was accessed to retrieve the MAF of the 19 prioritized variants in the general population. 18 of the prioritized variants were extremely rare in the global population (Table 3). In addition, all the variants have no ClinVar records with a defined classification of pathogenicity, indicating no ClinVar classification identified according to our search conditions (Table S12). M260I had no records in the gnomAD database.

### 3.6. Evolutionary Conservation Profile of RASSF2 Protein

**Figure 3:**
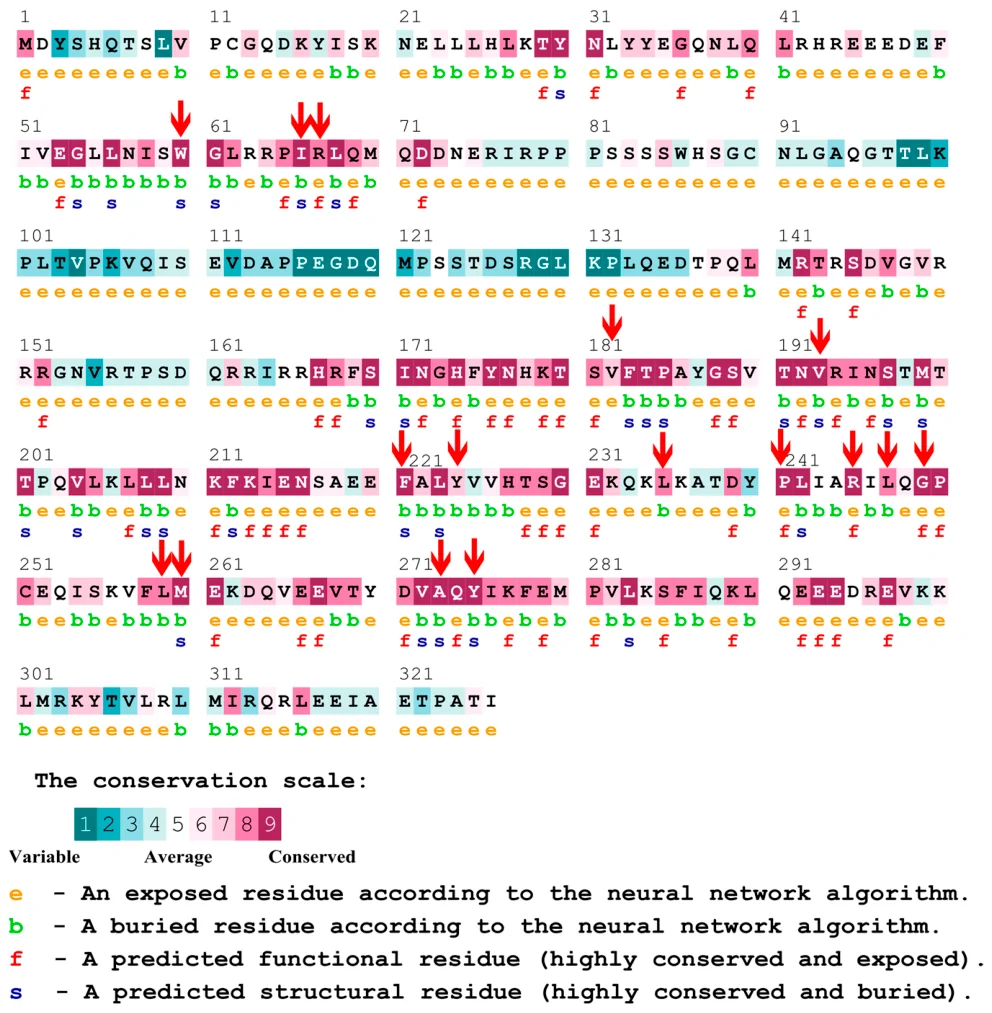
Conservation profile of the RASSF2 protein as generated by the ConSurf web server.

The conservation profile of RASSF2 was conducted using the ConSurf web server. The results showed that all 19 prioritized variants were conserved (score = 7–9). Of them, 13 variants were extremely conserved with a score of 9, four variants with a score of eight, and two variants with a score of 7 (Figure 3). InterPro enabled us to identify the domains and functional regions essential for the RASSF2 protein. Of the 19 variants, 14 variants were in the Ras-associating domain (RA domain), and two variants were in the SARAH domain (Table 4 and Table S13).

### 3.7. Prediction of Secondary Structure Differences Using NetSurfP 3.0

**Figure 4:**
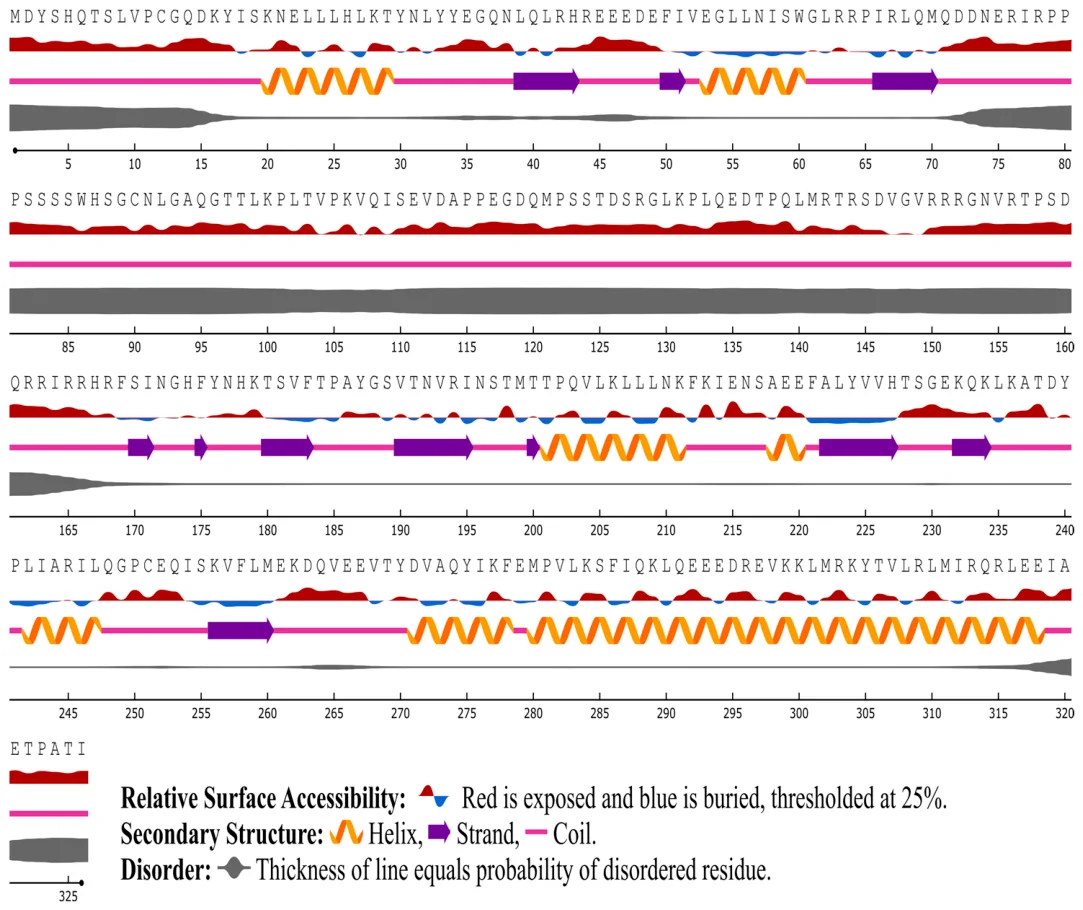
NetSurfP 3.0 prediction for the wild-type RASSF2 protein.

SS and solvent accessibility differences between the wild-type protein and the 19 variants were predicted using NetSurfP 3.0. NetSurfP predicted the α-helix, β-sheet, and coil regions of the protein (Figure 4) (Table S14). Predicted changes in SS were observed for W60R (α-helix to coil), L235P (Coil to β-sheet), and L247P (α-helix to coil). In addition, L247P showed an increase in RSA from 24% to 45%, exposing the buried residue. All the remaining 15 variants had the same SS, surface accessibility, and probability of disorder (Table 5).

### 3.8. Prediction of Cancer Driver Variants

All 19 variants were submitted to CScape to predict cancer-causing variants. All 19 variants were potentially oncogenic as predicted by CScape; of them, nine variants (W60R, R67S, V193F, V193D, L235P, R245Q, L247P, G249V, and A273T) were oncogenic with high confidence (Table 6). In addition, cBioPortal was accessed to investigate whether any of the variants were previously documented in cancer patient samples; of the 19 variants, R245Q was found in a colon adenocarcinoma sample. Additionally, several codon positions were found such as L235 (L235R in Lung Adenocarcinoma), P241 (P241R in Lung Adenocarcinoma), L247 (L247F in Uterine Endometrioid Carcinoma), and G249 (G249S in Uterine Endometrioid Carcinoma). We also accessed the AACR GENIE database, and we found out no data for RASSF2 is deposited in the database.

### 3.9. Prediction of Phosphorylated Sites

GPS 3.0 and NetPhos 3.1 servers were used to predict potential phosphorylated sites in the RASSF2 protein; out of the 19 prioritized variants, GPS 3.0 and NetPhos 3.1 predicted Y224C to be a phosphorylated site, while GPS 3.0 predicted Y275C to be a phosphorylated site (Table 7 and Table S15).

### 3.10. Prediction of Variants’ Molecular Effects Using MutPred2

MutPred2 was used to predict the molecular mechanism affected by the 19 destabilizing variants. The results showed that all 19 variants were predicted to be pathogenic (Score > 0.5), with some variants being predicted as pathogenic and highly confident (Score > 0.8). Also, molecular mechanisms affected were predicted for most variants except L247P and G249V. Out of all the 19 variants, four of them (W60R, V193D, L235P, and Y275C) are worth highlighting. W60R exhibited the highest g-score (0.945) among all variants, along with a predicted gain of intrinsic disorder (Probability = 0.54; *p*-value = 0.001). V193D exhibited a high g-score (0.93) along with a predicted alteration in the ordered interface (Probability = 0.43; *p*-value = 0.00035). L235P had a high g-score (0.896), with a predicted gain of intrinsic disorder (Probability = 0.34; *p*-value = 0.02). Y275C had a high g-score (0.833) and predicted loss of phosphorylation (Probability = 0.35; *p*-value = 0.01) (Table 8).

### 3.11. Prediction of Variants’ Structural Impact Using Project HOPE

Project HOPE analysis revealed various structural impacts of the 19 missense variants on protein structure. W60R was predicted to cause loss of hydrophobic interactions and repulsion of ligands or adjacent residues having the same charge. V193D was predicted to abolish the domain’s function and cause steric clashes due to the introduction of negative charge and increased size. Y224C was predicted to cause a loss of hydrogen bonds, thus disrupting protein folding. Interestingly, L247P was predicted to cause severe damage to the structure of the protein due to the introduction of proline, which breaks the α-helix structure. G249V might cause unusual torsion angles essential for the protein’s local conformation due to the substitution of glycine (Table 9).

### 3.12. Variants Impact on PPIs

**Figure 5:**
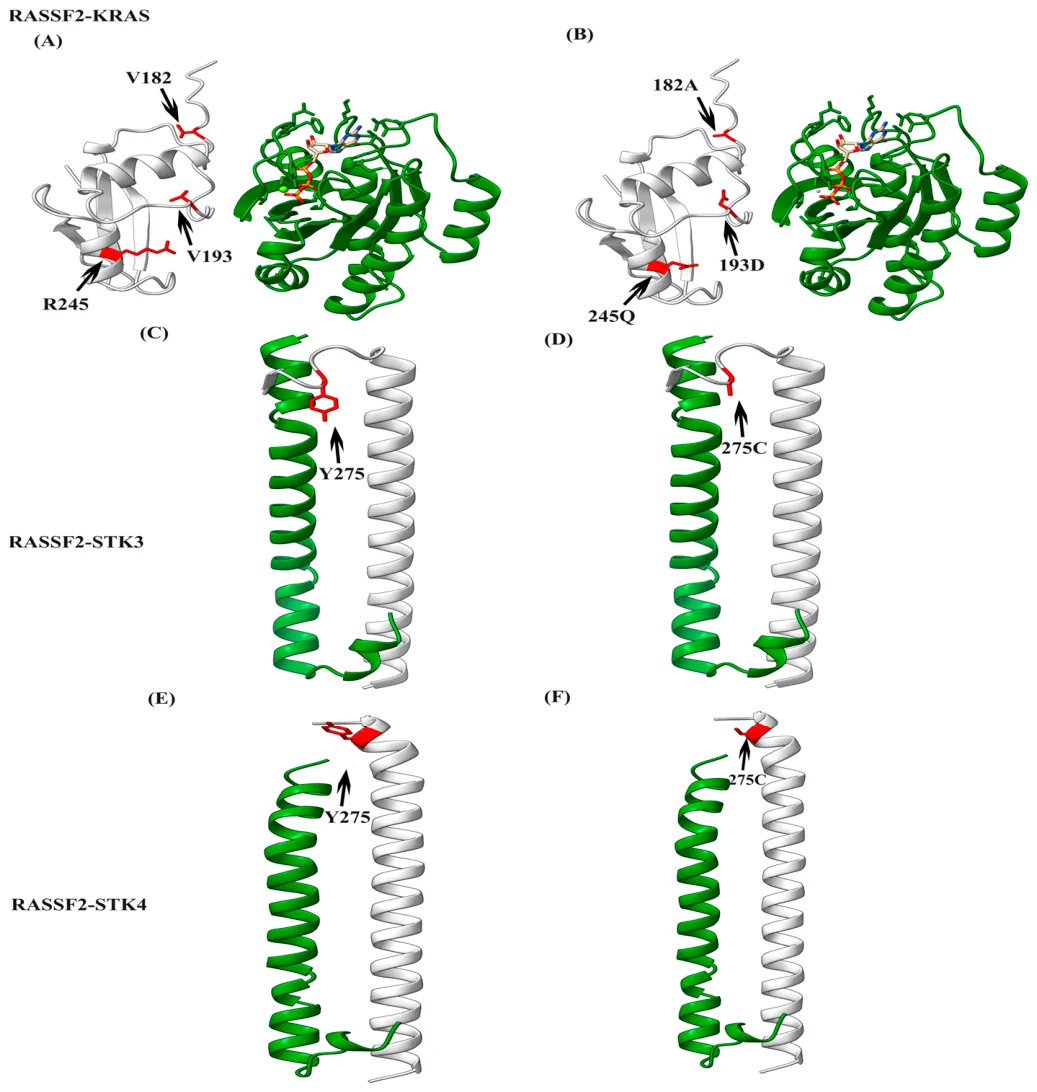
ChimeraX visualization of the protein complexes RASSF2-KRAS, RASSF2-STK3, and RASSF2-STK4 for V182A, V193D, R245Q, and Y275C variants colored in red. (**A**) represents wild-type RASSF2-KRAS, (**B**) represents mutant RASSF2-KRAS. (**C**) represents wild-type RASSF2-STK3, (**D**) represents mutant RASSF2-STK3. (**E**) represents wild-type RASSF2-STK4, (**F**) represents mutant RASSF2-STK4. The bound GTP ligand in the KRAS complex is displayed in stick representation colored by heteroatom (carbon in tan, nitrogen in blue, oxygen in red, and phosphorus in orange).

PPIs are essential for RASSF2 function; thus, predicting the impacts of missense variants on the interactions of RASSF2 with KRAS, MST1, and MST2 is important and increases the depth of our analysis. AlphaFold 3 was used to predict the complexes of both RASSF2-KRAS and RASSF2-MST1; pTM, ipTM, and PAE for each complex were assessed. For RASSF2-KRAS, the structure showed low-to-moderate (pTM = 0.63, ipTM = 0.52) interface confidence (Figure S1B). RASSF2-MST1 showed moderate (pTM = 0.68, ipTM = 0.66) interface confidence (Figure S1C). As for the RASSF2-MST2 structure retrieved from the AlphaFold database, it showed similar moderate (ipSAE: 0.73, ipTM: 0.68, pDockQ2: 0.62) interface confidence (Figure S1D) as RASSF2-MST1. Therefore, variants’ impact on PPIs should be interpreted with caution, specifically for the RASSF2-KRAS complex. Two tools (mCSM-PPI2 and MutPred-PPI) were used. mCSM-PPI2 predicted all variants to decrease binding affinity except A273T; MutPred-PPI predicted eight variants in the RASSF2-KRAS complex and one variant in the RASSF2-STK3 complex to be disruptive to the interactions. According to both tools, V193D and Y275C showed potential destabilization and disruption at the interface of the interactions of RASSF2-KRAS and RASSF2-MST2, respectively (Table 10). ChimeraX was used to visualize the protein complexes in wild-type vs. mutant forms for V182A, V193D, R245Q, and Y275C variants (Figure 5).

### 3.13. Correlation of Gene Expression and Mutations Using muTarget

**Figure 6:**
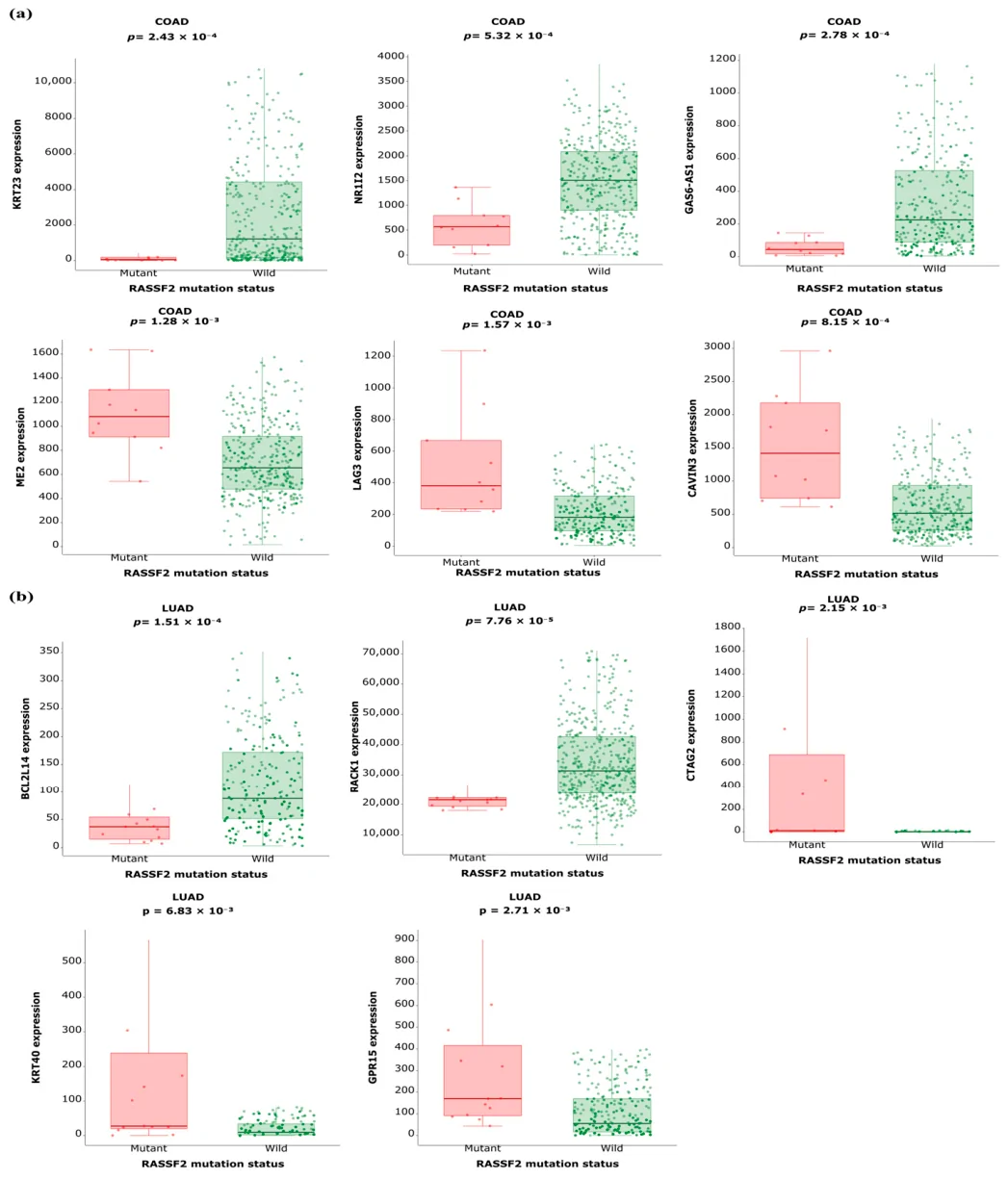
muTarget “Genotype” analysis for the RASSF2 gene. (**a**). Upregulated and downregulated genes due to coding mutations in the RASSF2 gene in COAD. (**b**). Upregulated and downregulated genes due to coding mutations in the RASSF2 gene in LUAD.

The muTarget tool enabled us to understand how coding variants affect gene expression. We used the ‘Genotype’ analysis, with RASSF2 being the mutated gene in two types of cancers, COAD and LUAD. In COAD, 217 genes were downregulated, while 31 genes were upregulated (Table S16). Mutations in RASSF2 showed absence of expression in the Keratin 23 (KRT23) gene, reduced expression in the Nuclear Receptor Subfamily 1 Group I-Member 2 (NR1I12) gene, and reduced expression in the Growth arrest specific 6 Antisense RNA 1 (GAS6-AS1) gene. Conversely, coding variants in RASSF2 showed significant upregulation in Malic Enzyme 2 (ME2), Lymphocyte Activating 3 (LAG3), and Caveolae Associated Protein 3 (CAVIN3). As for LUAD, 168 genes were downregulated, while 6 genes were upregulated (Table S16). Mutations in RASSF2 showed reduced expression in BCL2 Like 14 (BCL2L14) and receptor for activated C kinase 1 (RACK1). On the other hand, coding variants in RASSF2 showed significant upregulation in Cancer/Testis Antigen 2 (CTAG2), Keratin 40 (KRT40), and G Protein-Coupled Receptor 15 (GPR15) genes (Figure 6).

### 3.14. Protein–Protein Interactions and GO Enrichment Analysis

**Figure 7:**
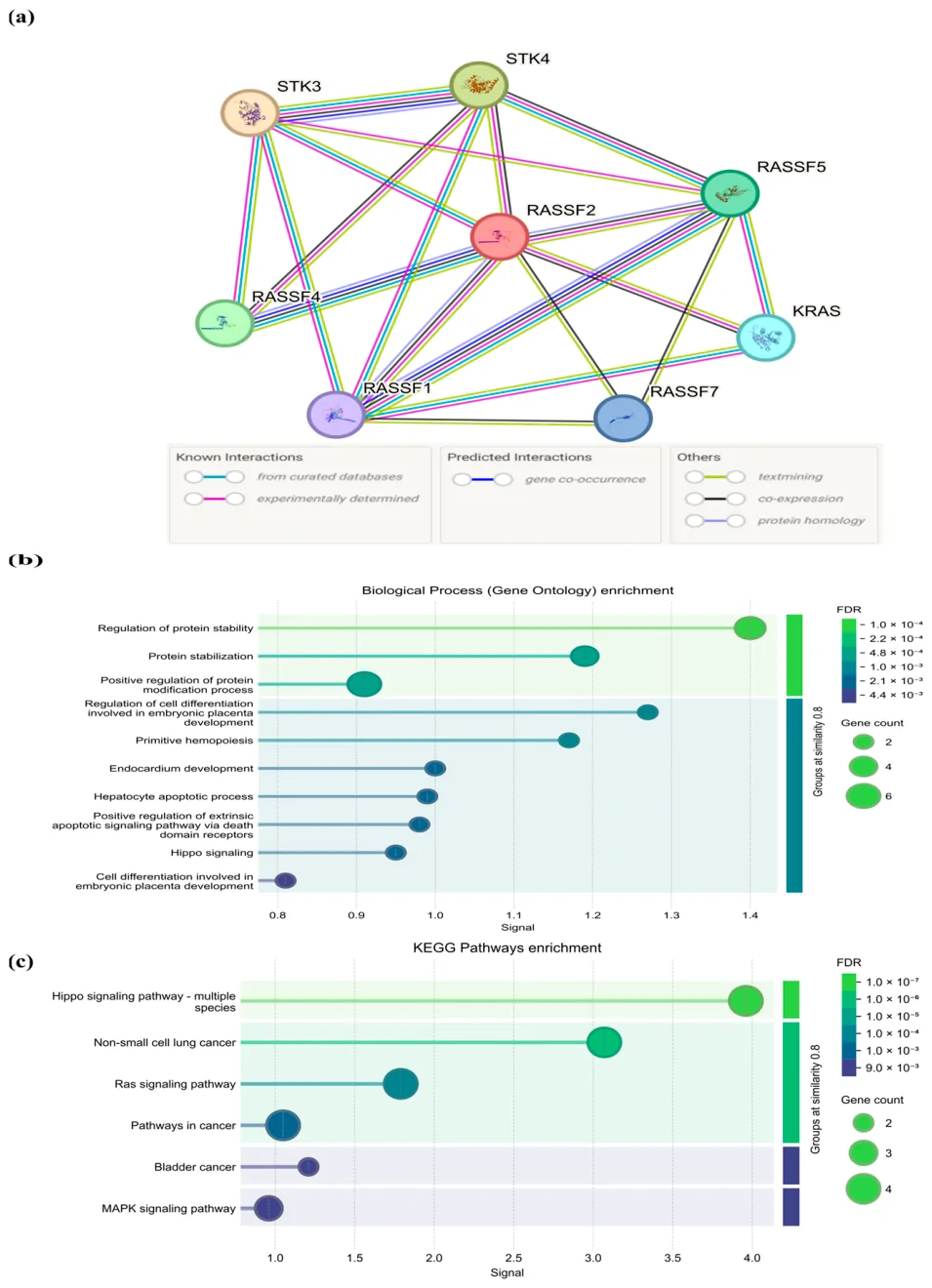
(**a**) PPI network constructed in the STRING tool. (**b**) GO biological process enrichment (**c**) KEGG pathway enrichment analysis.

STRING was used to predict the RASSF2 PPI network and related enrichment analysis. The network consists of 8 nodes and 19 edges, with a PPI enrichment *p*-value < 0.000149, indicating a significant enrichment. RASSF2 interacts with STK3 (score = 0.99), STK4 (score = 0.95), RASSF4 (score = 0.90), RASSF5 (0.89), KRAS (score = 0.821), RASSF7 (score = 0.77), and RASSF1 (score = 0.75) (Figure 7a).

GO biological process enrichment revealed regulation of protein stability, positive regulation of protein modification process, hepatocyte apoptotic process, and positive regulation of extrinsic apoptotic signaling pathway via death domain receptors (Figure 7b). KEGG pathway analysis illustrates the Hippo signaling pathway as the strongest enrichment pathway, non-small cell lung cancer (NSCLC), RAS signaling pathway, Bladder cancer, and MAPK signaling pathway (Figure 7c).

## 4. Discussion

RASSF2 is a tumor suppressor gene involved in multiple human cancers such as colon, lung, breast, and gastric cancer [50,51]. RASSF2 depends on the interaction of two domains, the RA and SARAH domains, for its function. RA domain binds directly to KRAS in a GTP-dependent manner via the Ras effector domain [2]. In SARAH, the Hippo signaling pathway is facilitated through the interaction of RASSF2 with two kinases, MST1 (STK4) and MST2 (STK3) [52]. Deletion of the RA domain impairs RASSF2’s ability to bind KRAS and therefore results in lower pro-apoptotic activity than the normal protein, indicating the importance of this domain for the protein function [53]. Missense variants may cause aberrant conformations, resulting in inactivation of its tumor suppressor activity [54,55]. Therefore, it is of utmost importance to investigate the impact of missense variants on RASSF2 and thereby their association with different diseases. In this work, we utilized a comprehensive in silico analysis to predict the most destructive missense variants and their impact on the structure and function of the RASSF2 protein.

Among 454 missense variants found in the NCBI dbSNP, 75 variants were predicted to be potentially pathogenic across all five prediction tools: SIFT, PolyPhen2, PhD-SNP^g^, E-SNPs&GO, and AlphaMissense. Using AlphaFold 3D structures, the impact of these variants on protein stability was predicted using DDMut, DynaMut2, and ThermoMPNN. As a result, 19 variants were predicted to be destabilizing (∆∆G < −0.5 in DDMut and DynaMut2; ∆∆G > 0.5 in ThermoMPNN) across the three tools. Protein stability is crucial for the function and structural integrity of the protein [56]. Alterations in a single amino acid sequence can have a profound effect on conformation and function, and may often lead to a disease [57]. It is worth noting that the excluded pathogenic variants might play a role in interaction specificity, regulatory sites, protein dynamics, localization, PTM, RNA splicing, and other mechanisms, but they were excluded for not fulfilling our destabilization criteria. Also. The 19 variants are algorithmically prioritized according to our selection pipeline, not necessarily meaning these are the only biologically important variants for RASSF2.

Next, we investigated the evolutionary conservation profile of the amino acid sequence using the ConSurf tool. We found that all 19 prioritized variants are conserved; specifically, 13 variants illustrate a highly conserved pattern with a score of 9. Usually, conserved regions in the protein are functionally important, and variants occurring in these regions are likely disease-causing variants [58]. Next, the InterPro tool was utilized to pinpoint the variants’ locations on the RASSF2 domains. Out of 19 variants, 16 variants were in functionally important domains for RASSF2. 14 variants were in the RA domain, and two variants were in the SARAH domain, an essential domain for RASSF2-induced apoptosis [10]. Such variants (A273T and Y275C) could have a major impact on the stability of the complex formed between RASSF2-MST1 and RASSF2-MST2.

Next, we investigated the secondary structure changes between the wild-type RASSF2 protein and its mutants using NetSurfP 3.0; we found that three variants exhibited changes in SS: W60R (α-helix to coil), L235P (coil to β-sheet), and L247P (α-helix to coil). SS elements are more conserved than the adjacent loops connecting them [59]. Mutations that disrupt them could have severe structural alterations that impair protein function and affect its binding to active GTP-bound KRAS. Also, L235P and L247P introduced proline, which forms a ring in the backbone and has a very rigid structure, and it can strongly impact the local backbone conformation, thus damaging the α-helix and β-sheet structures, although its structural consequences are context-dependent [60]. Furthermore, L247P was shown to change its buried status with exposure in the mutant proline; such a change might cause disruptions of structural integrity due to the loss of a buried residue [61].

CScape analysis predicted that all 19 variants are likely oncogenic; of them, nine variants were likely oncogenic with high confidence as predicted by CScape. Tumor suppressor genes play a pivotal role in the regulation of cellular functions, such as cell cycle arrest and induction of apoptosis [62]. These variants could resemble loss-of-function mutations, as they reside in crucial regions for protein function; such types of mutations have been found in multiple human cancers like lung and colorectal cancers [63,64,65]. In addition, multiple variant sites (235, 241, 245, 247, and 249) were found in patient samples from cBioPortal; this may suggest that this part of the RA segment is likely mutation-sensitive.

In addition, two variants (Y224C and Y275C) were predicted to harbor putative phosphorylation sites. Phosphorylation is a crucial PTM for proteins and is responsible for various biological processes such as regulation of protein activity, alteration of protein folding, and mediation or inhibition of PPIs [66]. Also, phosphorylation is important in cancer progression and cell proliferation [67]. Pathogenic mutations of human phosphorylation sites could have a profound effect on PPIs and might lead to disease [68].

MutPred2, which predicts the pathogenicity of variants along with affected molecular mechanisms, has found that all 19 variants were predicted pathogenic; 16 of them were associated with multiple molecular mechanisms. Five variants (W60R, I66T, R67S, V193D, and L235P) were associated with alterations in disorder; five variants (P241S, P241A, R245Q, A273T, and Y275C) were associated with alterations in PTM sites; and four variants (V182A, V193F, F221L, and Y224C) were associated with disruptions of structural components. Project HOPE further suggested how variants might disrupt the structure of the protein in terms of size, charge, hydrophobicity, and spatial organization. Several variants were predicted to damage the function of the domain due to the loss of important hydrophobic interactions required for protein stability. Three variants (R67S, Y224C, and Y275C) were predicted to cause loss of hydrogen bonds; such variants might disrupt the hydrogen bond network, leading to improper folding and stability, especially with Y224C and Y275C, since they are putative phosphorylation sites [69].

As mentioned above, the interaction between RASSF2 and KRAS in a GTP-dependent manner through the Ras effector domain, and the interaction between RASSF2 and MST1 (STK4) or MST2 (STK3) are crucial for its function; thus, predicting the impact of missense variants on these interactions is crucial. We utilized two tools, mCSM-PPI2 and MutPred-PPI. Out of 14 variants in the RA domain, mCSM-PPI2 predicted all 14 variants to decrease the binding affinity; notably, one variant (V193D) was predicted to decrease the binding affinity, and it was close to the domain interface. Also, in the SARAH domain, Y275C was predicted to decrease the binding affinity in both RASSF2-MST1 and RASSF2-MST2 complexes. Conversely, MutPred-PPI predicted eight variants to be disruptive in the RASSF2-KRAS complex, and one variant in the RASSF2-STK3 complex. These variants might cause loss of binding and stability, resulting in increased pathogenic potential [70]. Despite this, the results of these methods should be interpreted with caution, as the predicted AlphaFold 3 complexes are considered structural hypotheses, and that they predicted to have low to moderate confidence, specifically for the RASSF2-KRAS complex.

The muTarget tool enabled us to predict the impact of the target gene (RASSF2) coding mutations on the expression of other genes in COAD and LUAD patient samples. In COAD, our findings revealed lower expression in KRT23, NR1I2, and GAS6-AS1. KRT23 is a potential colorectal cancer biomarker that could activate human telomerase reverse transcriptase (hTERT) expression to promote CRC growth; thus, it could be targeted for therapy [71]. NRI12 (PXR) acts as a key regulator of xenobiotic metabolism in the liver, and it plays a role in CSC renewal and chemoresistance; thus, it may act as a promising target to minimize colorectal cancer relapse [72]. GAS6-AS1 can regulate GAS6 levels during the transcription or translation phase, resulting in boosting the AXL receptor tyrosine kinase (AXL) level and stimulating AXL signaling, and it can promote TRIM14-mediated cell proliferation, migration, invasion, and epithelial–mesenchymal transitions (EMTs) of CRC through a ceRNA network and in a FUS-dependent manner; thus, it could be considered a novel biomarker and a target for therapy [73]. Three upregulated genes were found (ME2, LAG3, CAVIN3). ME2 catalyzes the conversion of malate to pyruvate, and it was upregulated in different cancers, including colorectal cancer; thus, it can be targeted for therapy [74]. LAG3 acts as an inhibitory checkpoint receptor that was upregulated in various cancers, including CRC, and it may be a potential biomarker for prognosis [75]. CAVIN3 is considered a putative tumor suppressor gene [76]. muTarget indicated that CAVIN3 has higher expression than RASSF2; however, whether this represents a result of compensatory response or secondary downstream alterations remains an open hypothesis requiring functional validation. In LUAD, lower expression of BCL2L14 and RACK1 was predicted. BCL2L14 has pro-apoptotic activity; the lower expression of both RASSF2 and BCL2L14 could severely affect programmed cell death, making it easier for cancer cells to grow and resist treatments [77]. RACK1 is a scaffolding and ribosomal protein important in cell signaling pathways and the synthesis of proteins, with association with lung cancer growth [78]. Three genes were upregulated: CTAG2, KRT40, and GPR15. CTAG2 is a cancer/testis antigen abnormally expressed in breast cancer [79]. KRT40 is needed in building the epithelial cytoskeleton; the significant increase in expression could point to a specific epithelial reprogramming in lung cancer cells. GPR15 is a chemoattractant receptor that was documented in colon cancer by promoting a tumor-suppressive immune microenvironment [80].

STRING tool showed RASSF2 as a hub gene interacting with STK3/STK4, KRAS, and RASSF1, RASSF4, RASSF5, RASSF7. GO enrichment points toward protein stabilization, and the main pathway was found to be the Hippo signaling pathway. It is possible that some of the mutations predicted in our study could have a loss-of-function effect on RASSF2; thus, these mutations could perturb the intracellular and intercellular network, leading to diseases [81].

Although our bioinformatics pipeline played an essential role in the initial prioritization of nsSNPs affecting RASSF2, limitations must be acknowledged. Utilizing only computational methods, which are considered predictive methods, could limit our understanding of the impact of the predicted destabilizing nsSNPs on the protein structure and function. Experimental methods such as site-directed mutagenesis and protein stability assays are essential to confirm precision oncology utility. Also, PPI-predicted disruptions rely on predicted protein complexes that have low to moderate confidence, so these results must be interpreted with caution; the lack of experimental structures limits our ability to fully understand their impact on PPIs. In addition, muTarget findings represent general downstream correlation patterns, not a direct transcriptional effect from RASSF2.

## 5. Conclusions

RASSF2 is a tumor suppressor gene; its inactivation has been linked to multiple cancers. We systematically predicted the impact of missense variants on structure and function using an in silico pipeline. We found 19 candidate variants; 16 variants reside in the RA and SARAH domains, including V193D, L235P, L247P, and Y275C. These variants are key candidates that require experimental validation using site-directed mutagenesis or cellular assays to understand their role in cancer growth and metastasis. Also, population-based studies are essential to gain a better understanding of their impact.

## Figures and Tables

**Table 1:** Prediction of the most pathogenic variants using five tools.

		SIFT	PolyPhen 2	PhD-SNP	E-SNPs&GO	AlphaMissense
SNP ID	Variant	Score	Score	Score	Score	Score
rs200446635	Y30C	0.00	0.985	0.964	0.838	0.9733
rs1803854	N38I	0.01	0.999	0.916	0.708	0.6317
rs777839776	R42W	0.04	1	0.939	0.608	0.8233
rs138427842	R44W	0.05	1	0.882	0.624	0.7991
rs2514512856	G54V	0.00	0.999	0.888	0.933	0.9988
rs1446633611	L55R	0.01	1	0.900	0.608	0.978
rs761066545	W60R	0.00	0.978	0.976	0.891	0.9999
rs1370198314	W60L	0.00	0.989	0.983	0.891	0.9976
rs201330263	R63G	0.02	0.988	0.821	0.838	0.5667
rs745819966	R64L	0.00	0.916	0.862	0.719	0.9554
rs745819966	R64Q	0.00	0.999	0.904	0.474	0.6838
rs769557407	R64W	0.00	1	0.943	0.777	0.9303
rs1191523497	I66T	0.00	0.989	0.887	0.515	0.9991
rs147451876	R67H	0.03	1	0.943	0.515	0.7579
rs757099963	R67C	0.00	1	0.924	0.615	0.9016
rs757099963	R67S	0.03	1	0.752	0.708	0.9955
rs747554035	R142L	0.00	1	0.948	0.515	0.979
rs994162749	T143P	0.00	1	0.974	0.624	0.9066
rs2514482746	T143K	0.00	1	0.905	0.540	0.9616
rs746569074	R166P	0.03	1	0.945	0.584	0.9178
rs772769578	R168C	0.00	1	0.957	0.777	0.9486
rs772769578	R168G	0.00	1	0.962	0.860	0.9937
rs1328522470	R168H	0.00	1	0.961	0.719	0.924
rs1777844978	S170F	0.00	0.999	0.951	0.719	0.9972
rs1163515456	N172D	0.00	1	0.973	0.608	0.9956
rs1163515456	N172H	0.00	1	0.976	0.624	0.9719
rs1364825550	N172I	0.00	1	0.974	0.719	0.9983
rs779154515	G173D	0.00	1	0.944	0.891	0.9995
rs1048112272	G173S	0.00	1	0.944	0.860	0.9924
rs1925773040	H174R	0.03	0.999	0.972	0.615	0.9985
rs2514481714	H174Y	0.01	0.999	0.991	0.558	0.9936
rs1925696414	T180I	0.00	1	0.901	0.865	0.9988
rs2514477044	T180S	0.00	1	0.986	0.777	0.9896
rs2514477044	T180A	0.00	1	0.994	0.820	0.9966
rs1568564729	S181P	0.00	0.987	0.917	0.930	0.9988
rs188515817	V182M	0.03	1	0.972	0.540	0.9192
rs2514476888	V182A	0.02	0.994	0.995	0.558	0.9871
rs2514476854	F183L	0.02	0.999	0.938	0.820	1
rs1318938969	P185L	0.00	1	0.980	0.933	0.9967
rs1255956285	G188V	0.02	1	0.972	0.865	0.9943
rs1208560547	S189P	0.04	1	0.992	0.865	0.9987
rs1350526330	S189F	0.01	1	0.909	0.820	0.981
rs750014996	V193F	0.01	1	0.964	0.708	0.995
rs2514476531	V193D	0.00	1	0.978	0.865	0.9999
rs369276882	R194L	0.02	1	0.926	0.891	0.9913
rs778962328	R194C	0.00	1	0.914	0.860	0.9709
rs2514476408	S197N	0.00	1	0.972	0.708	0.9074
rs2514476419	S197G	0.00	0.999	0.966	0.624	0.7027
rs2514475918	K211N	0.00	1	0.626	0.865	0.9998
rs2514467641	E220V	0.02	0.991	0.916	0.515	0.9323
rs2514467571	F221L	0.00	0.999	0.891	0.708	0.9997
rs2514467590	F221S	0.00	1	0.880	0.860	0.9994
rs2514467518	Y224C	0.01	1	0.972	0.860	0.6289
rs1445343355	L235P	0.00	1	0.932	0.930	0.9977
rs1925487797	D239G	0.01	1	0.840	0.719	0.8934
rs769551649	P241L	0.00	1	0.972	0.930	0.9803
rs1266271957	P241S	0.00	1	0.981	0.891	0.9972
rs1266271957	P241A	0.00	1	0.916	0.820	0.9755
rs565787481	R245L	0.00	1	0.965	0.930	0.9753
rs565787481	R245Q	0.00	0.992	0.888	0.891	0.8648
rs765794600	L247P	0.00	0.999	0.973	0.930	0.988
rs2514463209	G249V	0.00	1	0.930	0.891	0.9957
rs1330224702	C251W	0.04	1	0.921	0.540	0.9409
rs760971921	L259R	0.00	1	0.984	0.955	0.9976
rs773121941	M260I	0.04	0.997	0.912	0.741	0.965
rs189107203	D271Y	0.00	1	0.972	0.933	0.963
rs189107203	D271H	0.00	1	0.623	0.891	0.9476
rs2514455122	V272E	0.01	1	0.978	0.719	0.9846
rs2514455083	A273T	0.01	1	0.888	0.474	0.9951
rs1485852692	Y275C	0.00	1	0.947	0.933	0.9976
rs2514454950	I276T	0.03	0.999	0.897	0.558	0.9851
rs770738940	P281L	0.01	1	0.954	0.654	0.9385
rs778413517	L290P	0.00	1	0.928	0.953	0.9996
rs2122415340	K304M	0.00	0.833	0.732	0.515	0.9275
rs2514442979	Y305H	0.00	0.948	0.825	0.515	0.9895

SIFT: A score less than 0.05 indicates potentially deleterious variants. PolyPhen 2: A score higher than 0.5 indicates likely damaging variants. PhD-SNP: A score higher than 0.5 indicates a predicted pathogenic variant. E-SNPs&GO: A score higher than 0.5 indicates a predicted pathogenic variant. AlphaMissense: A score higher than 0.5 indicates a likely pathogenic variant.

**Table 2:** Variants predicted to be destabilizing in DDMut, DynaMut2, and ThermoMPNN.

			DDMut	DynaMut 2	ThermoMPNN
SNP ID	Variant	plDDT	∆∆G	∆∆G	∆∆G
rs761066545	W60R	97.31	−1.72	−1.56	3.77
rs1191523497	I66T	93.50	−2.02	−2.83	2.19
rs757099963	R67S	92.50	−0.96	−0.52	1.33
rs2514476888	V182A	91.19	−0.83	−1.78	1.07
rs750014996	V193F	97.25	−0.89	−1.49	0.7
rs2514476531	V193D	97.25	−2.35	−2.62	2.38
rs2514467571	F221L	96.62	−0.88	−1.88	1.18
rs2514467590	F221S	96.62	−2.85	−3.75	2.31
rs2514467518	Y224C	98.12	−3.24	−2.22	2.6
rs1445343355	L235P	97.38	−2.69	−1.96	2.33
rs1266271957	P241S	98.19	−2.47	−3.48	1.9
rs1266271957	P241A	98.19	−1.58	−1.81	1.21
rs565787481	R245Q	97.75	−1.81	−1.18	1.03
rs765794600	L247P	96.81	−1.34	−0.91	2.38
rs2514463209	G249V	96.25	−0.93	−0.66	1.86
rs760971921	L259R	97.94	−2.03	−1.82	2.96
rs773121941	M260I	97.69	−1.02	−0.57	0.98
rs2514455083	A273T	93.75	−0.75	−0.7	1.76
rs1485852692	Y275C	93.44	−0.56	−0.57	0.92

∆∆G < −0.5 indicates a predicted destabilizing variant in DDMut, DynaMut2. ∆∆G > 0.5 indicates a predicted destabilizing variant in ThermoMPNN. A plDDT > 90 indicates very high confidence.

**Table 3:** gnomAD data for the allele frequency for the prioritized variants.

SNP ID	Variant	Allele Count/Number	Allele Frequency	Genetic Ancestry
rs761066545	W60R	11/1579606	6.96 × 10^−6^	8-NFE, 2-admixed American, 1 unknown
rs1191523497	I66T	2/1606208	1.25 × 10^−6^	1-NFE, 1-SA
rs757099963	R67S	1/1606684	6.22 × 10^−7^	1-NFE
rs2514476888	V182A	1/1613980	6.20 × 10^−7^	1-NFE
rs750014996	V193F	1/1613908	6.20 × 10^−7^	1-AFR/AMR
rs2514476531	V193D	1/1614034	6.20 × 10^−7^	1-NFE
rs2514467571	F221L	1/1609090	6.21 × 10^−7^	1-AFR/AMR
rs2514467590	F221S	1/1609198	6.21 × 10^−7^	1-NFE
rs2514467518	Y224C	1/1607568	6.22 × 10^−7^	1-NFE
rs1445343355	L235P	1/1614008	6.20 × 10^−7^	1-NFE
rs1266271957	P241S	1/1614140	6.20 × 10^−7^	1-EA
rs1266271957	P241A	1/1614140	6.20 × 10^−7^	1-SA
rs565787481	R245Q	16/1613994	9.91 × 10^−6^	10-NFE, 6-SA
rs765794600	L247P	2/1614110	1.24 × 10^−6^	2-NFE
rs2514463209	G249V	1/1614136	6.20 × 10^−7^	1-NFE
rs760971921	L259R	1/1614138	6.20 × 10^−7^	1-SA
rs2514455083	A273T	1/1600822	6.25 × 10^−7^	1-NFE
rs1485852692	Y275C	2/1607006	1.24 × 10^−6^	2-NFE

AFR/AMR = African/American; NFE = non-Finnish European; SA = South Asian; EA = East Asian.

**Table 4:** Conservation score and domain identification of the RASSF2 protein.

SNP ID	Variant	Conservation	InterPro Domains
rs761066545	W60R	Buried, structural, and highly conserved residue (9)	NA
rs1191523497	I66T	Buried, structural, and highly conserved residue (9)	NA
rs757099963	R67S	Exposed, functional, and conserved residue (8)	NA
rs2514476888	V182A	Exposed and conserved residue (7)	RA domain
rs750014996	V193F	Buried, structural, and highly conserved residue (9)	RA domain
rs2514476531	V193D	Buried, structural, and highly conserved residue (9)	RA domain
rs2514467571	F221L	Buried, structural, and highly conserved residue (9)	RA domain
rs2514467590	F221S	Buried, structural, and highly conserved residue (9)	RA domain
rs2514467518	Y224C	Buried and conserved residue (7)	RA domain
rs1445343355	L235P	Buried and conserved residue (8)	RA domain
rs1266271957	P241S	Exposed, functional, and highly conserved residue (9)	RA domain
rs1266271957	P241A	Exposed, functional, and highly conserved residue (9)	RA domain
rs565787481	R245Q	Exposed, functional, and highly conserved residue (9)	RA domain
rs765794600	L247P	Buried and conserved residue (8)	RA domain
rs2514463209	G249V	Exposed, functional, and highly conserved residue (9)	RA domain
rs760971921	L259R	Buried and conserved residue (8)	RA domain
rs773121941	M260I	Buried, structural, and highly conserved residue (9)	RA domain
rs2514455083	A273T	Buried, structural, and highly conserved residue (9)	SARAH domain
rs1485852692	Y275C	Buried, structural, and highly conserved residue (9)	SARAH domain

**Table 5:** NetSurfP prediction for SS, RSA, ASA, and Pdisorder between the wild-type and the 19 prioritized variants.

SNP ID	Variant	WT vs. Mut	Class	SS	RSA	ASA	Pdisorder
rs761066545	W60R	WT	Buried	α-helix	12%	35 Å^2^	1%
		Mut	Buried	Coil	24%	67 Å^2^	1%
rs1191523497	I66T	WT	Buried	β-sheet	3%	5 Å^2^	0%
		Mut	Buried	β-sheet	6%	9 Å^2^	1%
rs757099963	R67S	WT	Exposed	β-sheet	31%	86 Å^2^	1%
		Mut	Exposed	β-sheet	26%	41 Å^2^	2%
rs2514476888	V182A	WT	Buried	β-sheet	13%	21 Å^2^	1%
		Mut	Buried	β-sheet	22%	29 Å^2^	1%
rs750014996	V193F	WT	Buried	β-sheet	3%	5 Å^2^	0%
		Mut	Buried	β-sheet	4%	11 Å^2^	0%
rs2514476531	V193D	WT	Buried	β-sheet	3%	5 Å^2^	0%
		Mut	Buried	β-sheet	3%	7 Å^2^	0
rs2514467571	F221L	WT	Buried	Coil	4%	10 Å^2^	0%
		Mut	Buried	Coil	5%	10 Å^2^	0%
rs2514467590	F221S	WT	Buried	Coil	4%	10 Å^2^	0%
		Mut	Buried	Coil	5%	8 Å^2^	0%
rs2514467518	Y224C	WT	Buried	β-sheet	6%	15 Å^2^	0%
		Mut	Buried	β-sheet	5%	8 Å^2^	0%
rs1445343355	L235P	WT	Buried	Coil	7%	14 Å^2^	0%
		Mut	Buried	β-sheet	9%	15 Å^2^	0%
rs1266271957	P241S	WT	Buried	Coil	8%	13 Å^2^	0%
		Mut	Buried	Coil	16%	25 Å^2^	0%
rs1266271957	P241A	WT	Buried	Coil	8%	13 Å^2^	0%
		Mut	Buried	Coil	18%	14 Å^2^	0%
rs565787481	R245Q	WT	Exposed	α-helix	28%	76 Å^2^	0%
		Mut	Exposed	α-helix	33%	74 Å^2^	0%
rs765794600	L247P	WT	Buried	α-helix	24%	48 Å^2^	0%
		Mut	Exposed	Coil	45%	71 Å^2^	1%
rs2514463209	G249V	WT	Exposed	Coil	28%	29 Å^2^	2%
		Mut	Exposed	Coil	33%	57 Å^2^	2%
rs760971921	L259R	WT	Buried	β-sheet	4%	8 Å^2^	0%
		Mut	Buried	β-sheet	11%	30 Å^2^	0%
rs773121941	M260I	WT	Buried	β-sheet	15%	33 Å^2^	0%
		Mut	Buried	β-sheet	13%	26 Å^2^	0%
rs2514455083	A273T	WT	Buried	α-helix	10%	13 Å^2^	1%
		Mut	Buried	α-helix	20%	34 Å^2^	1%
rs1485852692	Y275C	WT	Buried	α-helix	10%	27 Å^2^	0%
		Mut	Buried	α-helix	10%	16 Å^2^	0%

RSA higher than 25% indicates an exposed site.

**Table 6:** Prediction of oncogenic variants using CScape.

		CScape	
SNP ID	Variant	Prediction	Score
rs761066545	W60R	Oncogenic (High confidence)	0.9
rs1191523497	I66T	Oncogenic	0.87
rs757099963	R67S	Oncogenic (High confidence)	0.91
rs2514476888	V182A	Oncogenic	0.86
rs750014996	V193F	Oncogenic (High confidence)	0.89
rs2514476531	V193D	Oncogenic (High confidence)	0.91
rs2514467571	F221L	Oncogenic	0.67
rs2514467590	F221S	Oncogenic	0.84
rs2514467518	Y224C	Oncogenic	0.88
rs1445343355	L235P	Oncogenic (High confidence)	0.94
rs1266271957	P241S	Oncogenic	0.88
rs1266271957	P241A	Oncogenic	0.88
rs565787481	R245Q	Oncogenic (High confidence)	0.97
rs765794600	L247P	Oncogenic (High confidence)	0.96
rs2514463209	G249V	Oncogenic (High confidence)	0.9
rs760971921	L259R	Oncogenic	0.82
rs773121941	M260I	Oncogenic	0.70
rs2514455083	A273T	Oncogenic (High confidence)	0.91
rs1485852692	Y275C	Oncogenic	0.89

A probability > 0.5 indicates an oncogenic variant in CScape, while a probability > 0.9 indicates high-confidence predictions.

**Table 7:** Variants predicted to disrupt phosphorylated sites.

SNP ID	Variant	PTM	Prediction Score
rs2514467518	Y224C	Phosphorylated site—GPS 3.0 and NetPhos 3.1	0.59 (NetPhos 3.1). 0.77 (GPS 3.0)
rs1485852692	Y275C	Phosphorylated site—GPS 3.0	0.78 (GPS 3.0)

A score > 0.5 indicates a predicted phosphorylated site in NetPhos 3.1. A score > 0.76 indicates a predicted phosphorylated site in GPS 3.0.

**Table 8:** MutPred2 prediction for the 19 prioritized variants.

SNP ID	Variant	MutPred2 Score	Predicted Molecular Mechanisms
rs761066545	W60R	0.945	Gain of Intrinsic disorder (Pr = 0.54|*p* = 0.001);
			Altered Disordered interface (Pr = 0.19|*p* = 0.03);
			Altered DNA binding (Pr = 0.15|*p* = 0.04);
			Altered Transmembrane protein (Pr = 0.10|*p* = 0.04);
			Loss of N-linked glycosylation at N57 (Pr = 0.04|*p* = 0.03)
rs1191523497	I66T	0.88	Gain of Intrinsic disorder (Pr = 0.35|*p* = 0.02);
			Altered Disordered interface (Pr = 0.34|*p* = 0.01);
			Altered DNA binding (Pr = 0.18|*p* = 0.03)
rs757099963	R67S	0.863	Altered Disordered interface (Pr = 0.29|*p* = 0.03);
			Loss of Helix (Pr = 0.27|*p* = 0.04);
			Altered DNA binding (Pr = 0.18|*p* = 0.03)
rs2514476888	V182A	0.762	Loss of Strand (Pr = 0.27|*p* = 0.02);
			Altered Transmembrane protein (Pr = 0.12|*p* = 0.03);
			Altered Stability (Pr = 0.11|*p* = 0.03)
rs750014996	V193F	0.879	Gain of Strand (Pr = 0.26|*p* = 0.04);
			Altered DNA binding (Pr = 0.18|*p* = 0.03);
			Altered Metal binding (Pr = 0.11|*p* = 0.04);
			Altered Transmembrane protein (Pr = 0.10|*p* = 0.04);
			Altered Stability (Pr = 0.10|*p* = 0.04);
			Loss of N-linked glycosylation at N196 (Pr = 0.05|*p* = 0.02)
rs2514476531	V193D	0.93	Altered Ordered interface (Pr = 0.43|*p* = 0.00035);
			Altered Stability (Pr = 0.22|*p* = 0.01);
			Altered DNA binding (Pr = 0.21|*p* = 0.02);
			Altered Metal binding (Pr = 0.20|*p* = 0.04);
			Altered Transmembrane protein (Pr = 0.12|*p* = 0.03);
			Loss of N-linked glycosylation at N196 (Pr = 0.05|*p* = 0.02)
rs2514467571	F221L	0.869	Gain of Helix (Pr = 0.29|*p* = 0.02);
			Loss of Strand (Pr = 0.28|*p* = 0.008)
rs2514467590	F221S	0.914	Gain of B-factor (Pr = 0.25|*p* = 0.03);
			Altered Stability (Pr = 0.09|*p* = 0.05)
rs2514467518	Y224C	0.871	Gain of Helix (Pr = 0.27|*p* = 0.04);
			Loss of Strand (Pr = 0.27|*p* = 0.01)
rs1445343355	L235P	0.896	Gain of Intrinsic disorder (Pr = 0.34|*p* = 0.02);
			Gain of B-factor (Pr = 0.28|*p* = 0.0062);
			Altered Stability (Pr = 0.11|*p* = 0.03);
			Gain of Sulfation at Y240 (Pr = 0.02|*p* = 0.03)
rs1266271957	P241S	0.802	Loss of Sulfation at Y240 (Pr = 0.02|*p* = 0.04)
rs1266271957	P241A	0.743	Gain of Sulfation at Y240 (Pr = 0.02|*p* = 0.04)
rs565787481	R245Q	0.708	Gain of Sulfation at Y240 (Pr = 0.03|*p* = 0.02)
rs765794600	L247P	0.78	NA
rs2514463209	G249V	0.772	NA
rs760971921	L259R	0.907	Altered Stability (Pr = 0.26|*p* = 0.0072)
rs773121941	M260I	0.526	NA
rs2514455083	A273T	0.769	Gain of Acetylation at K277 (Pr = 0.19|*p* = 0.04);
			Loss of Proteolytic cleavage at D271 (Pr = 0.12|*p* = 0.03);
			Altered Coiled coil (Pr = 0.08|*p* = 0.05);
			Gain of Sulfation at Y270 (Pr = 0.03|*p* = 0.02)
rs1485852692	Y275C	0.833	Loss of Phosphorylation at Y275 (Pr = 0.35|*p* = 0.01);
			Altered Ordered interface (Pr = 0.24|*p* = 0.04);
			Loss of Acetylation at K277 (Pr = 0.23|*p* = 0.02);
			Loss of Proteolytic cleavage at D271 (Pr = 0.12|*p* = 0.03);
			Altered Coiled coil (Pr = 0.08|*p* = 0.05);
			Loss of Sulfation at Y270 (Pr = 0.03|*p* = 0.02)

A g-score > 0.5 indicates a predicted pathogenic variant.

**Table 9:** Project HOPE prediction for the structural impact of variants on the protein.

SNP ID	Variant	Project HOPE Report
rs761066545	W60R	The difference in charge might cause repulsion of ligands or other residues with the same charge, along with potential loss of hydrophobic interactions.
rs1191523497	I66T	The difference in size and hydrophobicity between the WT and mutant might cause the loss of hydrophobic interactions either in the core of the protein or on the surface.
rs757099963	R67S	The difference in charge might cause a loss of interactions with other residues. Additionally, the difference in size and hydrophobicity might cause the loss of H-bonds and disruptions of protein folding.
rs2514476888	V182A	The variant might abolish the function of the domain and cause loss of interactions due to size differences.
rs750014996	V193F	The variant might abolish the function of the domain and cause the introduction of steric clashes due to its larger size.
rs2514476531	V193D	Because of negative charge introduction and size differences, the variant might abolish the function of the domain, introduce steric clashes, and cause repulsion of ligands.
rs2514467571	F221L	The variant might abolish the function of the domain and cause loss of interactions due to its smaller size.
rs2514467590	F221S	The variant might abolish the function of the domain, cause loss of interactions due to its smaller size, and cause loss of hydrophobic interactions due to hydrophobicity change.
rs2514467518	Y224C	The variant might abolish the function of the domain. Additionally, the difference in size and hydrophobicity might cause the loss of H-bonds and disruptions of protein folding.
rs1445343355	L235P	The variant might abolish the function of the domain and cause loss of interaction due to size change.
rs1266271957	P241S	The variant might abolish the function of the domain and cause the loss of hydrophobic interactions. Additionally, proline has a very rigid structure; such a loss of proline might cause the loss of the special backbone conformation.
rs1266271957	P241A	The variant might abolish the function of the domain and cause the loss of backbone conformation due to proline loss.
rs565787481	R245Q	The variant might abolish the function of the domain and cause loss of interactions due to its smaller size. Also, the loss of charge might disrupt interactions with other molecules or residues.
rs765794600	L247P	Since the wild type is in an α-helix, and proline breaks the α-helix, this variant might cause a severe effect on the structure of the protein.
rs2514463209	G249V	Since the wild type is the most flexible amino acid, glycine, variation at this critical domain might abolish its function, and the unusual torsion angles caused by glycine might disrupt the local conformation
rs760971921	L259R	The variant might abolish the function of the domain and cause the introduction of steric clashes due to its larger size. Also, the introduced positive charge might cause repulsion of ligands.
rs773121941	M260I	This variant might cause a possible loss of external interaction as the mutant residue is smaller than the wild-type residue.
rs2514455083	A273T	The variant might abolish the function of the domain and cause steric clashes.
rs1485852692	Y275C	The variant might abolish the function of the domain. Additionally, the difference in size and hydrophobicity might cause the loss of H-bonds and disruptions of protein folding.

**Table 10:** Predicted Impact of missense variants on PPIs using mCSM-PPI2 and MutPred-PPI.

				mCSM-PPI2			MutPred-PPI	
Protein	AF Metrics	Variant	AF3 plDDT	Distance-to-Interface	∆∆G	Prediction	Score	Prediction
KRAS	pTM: 0.63 ipTM: 0.52	V182A	very low confident	7.777	−0.198	Decrease	0.1561	Preserved
		V193F	low confident	4.081	−0.163	Decrease	0.4937	Preserved
		V193D	low confident	4.081	−0.697	Decrease	0.8325	Disrupted
		F221L	Confident	12.394	−0.614	Decrease	0.5168	Disrupted
		F221S	Confident	12.394	−0.302	Decrease	0.9106	Disrupted
		Y224C	Confident	15.15	−0.177	Decrease	0.4274	Preserved
		L235P	low confident	16.566	−0.904	Decrease	0.7752	Disrupted
		P241S	low confident	13.258	−0.252	Decrease	0.8375	Disrupted
		P241A	low confident	13.258	−0.336	Decrease	0.6716	Disrupted
		R245Q	low confident	7.746	−0.236	Decrease	0.2928	Preserved
		L247P	low confident	17.261	−0.576	Decrease	0.5744	Disrupted
		G249V	low confident	12.017	−0.292	Decrease	0.4	Preserved
		L259R	low confident	10.607	−0.432	Decrease	0.7059	Disrupted
		M260I	low confident	12.358	−0.221	Decrease	0.399	Preserved
MST1	pTM: 0.68	A273T	Confident	8.545	0.078	Increase	0.332	Preserved
	ipTM: 0.66	Y275C	Confident	5.305	−0.435	Decrease	0.343	Preserved
MST2	ipSAE: 0.73 (confident)	A273T	Very high Confident	3.248	0.575	Increase	0.259	Preserved
	ipTM: 0.68 pDockQ2: 0.62	Y275C	Very high Confident	3.525	−2.726	Decrease	0.563	Disrupted

## Data Availability

All data used in our study are available in the Supplementary Materials.

## Supplementary Materials

The following supporting information can be downloaded at: https://www.mdpi.com/article/10.3390/biology15201818/s1, Table S1: Missense variants retrieved from the NCBI dbSNP; Table S2: SIFT prediction results for 454 variants; Table S3: PolyPhen2 prediction results for 454 variants; Table S4: PhD-SNP prediction results for 454 variants; Table S5: E-SNPs&GO prediction results for 454 variants; Table S6: AlphaMissense prediction results for missense variants; Table S7: Consensus table results for all five tools; Table S8: DDMut prediction results for structural stability due to variants; Table S9: DynaMut2 prediction results for structural stability due to variants; Table S10: ThermoMPNN prediction results for structural stability due to variants; Table S11: Consensus table results for all three stability tools; Table S12: gnomAD results for RASSF2 variants; Table S13: InterPro results for RASSF2 protein; Table S14: NetSurfP 3.0 results for RASSF2 protein; Table S15: NetPhos 3.1 and GPS 6.0 results for putative phosphorylation sites; Table S16: muTarget results for upregulated and downregulated genes affected from RASSF2 coding mutations. Figure S1: PAE plots for the generated protein structures. (A). PAE plot for the wild-type RASSF2 protein structure. (B). PAE plot for the predicted RASSF2-KRAS complex. (C). PAE plot for the predicted RASSF2-MST1 complex. (D). PAE plot for the predicted RASSF2-MST2 complex.

## Abbreviations

The following abbreviations are used in this manuscript:

RASSF2	Ras association (RalGDS/AF-6) domain family member 2
SARAH	Sav/Rassf/Hpo domain
PPIs	Protein–protein interactions
nsSNPs	Non-synonymous single nucleotide polymorphisms
NCBI	National Center for Biotechnology Information
SIFT	The Sorting Intolerant from Tolerant
PolyPhen2	The Polymorphism Phenotyping v2
3D	Three-dimensional
pLDDT	Predicted Local Distance Difference Test
pTM	Predicted template modelling
GTP	Guanosine triphosphate
Mg^2+^	Magnesium ions
ΔΔG	Gibbs Free Energy
PDB	Protein Data Bank
gnomAD	Genome Aggregation Database
MAF	Minor allele frequencies
SS	Secondary structures
Pdisorder	Probability of disorder
RSA	Relative surface accessibility
ASA	Absolute surface accessibility
PK	Protein kinase
Project HOPE	Have (y) Our Protein Explained
pLMs	Protein language models
AUC	Area under the curve
COAD	Colon adenocarcinoma
LUAD	Lung adenocarcinoma
STRING	Search Tool for the Retrieval of Interacting Genes/Proteins
GO	Gene Ontology
KEGG	Kyoto Encyclopedia of Genes and Genomes
KRT23	Keratin 23
NR1I12	Nuclear Receptor Subfamily 1 Group I-Member 2
GAS6-AS1	Growth arrest specific 6 Antisense RNA 1
ME2	Malic Enzyme 2
LAG3	Lymphocyte Activating 3
CAVIN3	Caveolae Associated Protein 3
BCL2L14	BCL2-like 14
RACK1	Receptor for activated C kinase 1
CTAG2	Cancer/Testis Antigen 2
KRT40	Keratin 40
GPR15	G Protein-Coupled Receptor 15
NSCLC	Non-small cell lung cancer
