# Supplementary material for: RASSF2 Under the Computational Microscope: Prediction of Destabilizing Missense Variants
Source: Biology (Basel). 2026 Oct 12;15(20):1818. doi: 10.3390/biology15201818 (PMC13701346; doi:10.3390/biology15201818)
Supplement: Supplementary file 1 [file biology-15-01818-s001.zip › Figure S1.pdf]

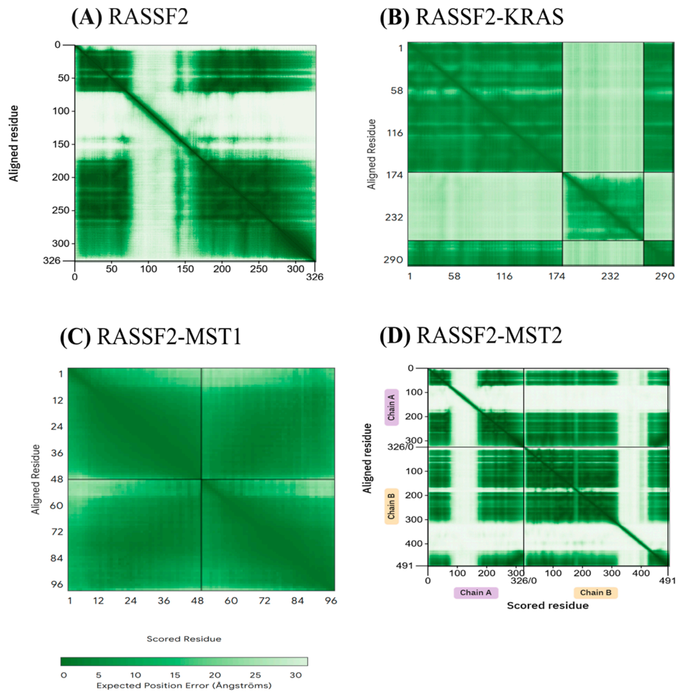

Figure S1: PAE plots for the generated protein structures. (A). PAE plot for the wild-type RASSF2 protein structure. (B). PAE plot for the predicted RASSF2-KRAS complex. (C). PAE plot for the predicted RASSF2-MST1 complex. (D). PAE plot for the predicted RASSF2-MST2 complex.
